# Provincial Medical Schools

**Published:** 1898-09-03

**Authors:** 


### PROVINCIAL MEDICAL SCHOOLS.

At all the provincial medical schools the course of study can be so arranged as to allow of a degree being taken at the London University, and just as in regard to London students, arrangements can be made for residing a year at Newcastle so as to qualify for the Durham degree, or to reside two years at one or other of the Universities of Scotland so as to graduate there. Certain of the provincial schools, however, are directly connected with degree-giving Universities, namely, those at Oxford, Cambridge, and Newcastle (Durham); and those connected with the Victoria University, namely, Leeds (Yorkshire College), Liverpool (University College), and Manchester (Owens College). Those at Birmingham (Mason University College), Bristol (University College), and Sheffield (University College), and also the medical school at Cardiff, which at present provides only for the first portion of the curriculum, do not at present form an integral part of any university.

Students at Oxford and Cambridge as a rule, and those from Cardiff always, do their hospital work in London or some large town. The other schools in the list provide for the entire curriculum.

It is to be noted that all the provincial medical schools are faculties or departments of large colleges, or, as at Oxford and Cambridge, are schools connected with universities. They are not mere appendages to the hospitals, as is often the case in London, and thus, in many cases, the fees for the hospital practice and the college courses are separate.

### BIRMINGHAM.

Mason University College: Queen's Faculty of Medicine.

The General Hospital and the Queen's Hospital, in both of which clinical instruction is given, together contain over 400 beds, and receive 6,000 in-patients in the year.

The composition college fee is £70 on entrance, or in two annual instalments; that for the hospital practice is £42.

Students have also free admission to the City Lunatic Asylum and several special hospitals.

### BRISTOL.

University College: Faculty of Medicine.

The Royal Infirmary contains 264 beds, and receives about 3,201 in-patients in the year; and the General Hospital contains 200 beds, and receives about 2,333 in-patients in the year.

The composition fee is 65 guineas for the college course, and 35 guineas for the hospital practice.

### CAMBRIDGE.

In connection with the University course hospital practice can be followed at the Addenbrooke's Hospital, which has 158 beds, and receives 1,348 in-patients in the year. The hospital fee is £8 8s.

### CARDIFF.

University College: Medical School.

This school provides for the first portion of the curriculum, including the preliminary scientific and the intermediate examination for the London M.B., and the first and second examination of the Conjoint Board. For the latter the composition fee is £35.

### LEEDS.

The Yorkshire College: Medical Department.

The Leeds General Infirmary has about 400 beds, and receives 6,116 in-patients in the year.

The composition fee is £73 10s. for the college, and £48 6s. for the hospital, including the Fever Hospital, obstetric course, and vaccination.

### LIVERPOOL.

University College: Medical Faculty.

The Royal Infirmary contains 300 beds, and 3,009 in-patients have been received in the year. The practice of several special hospitals in the immediate vicinity is also open to the students of the medical faculty. The new laboratories, which have been erected and equipped by the Rev. S. A. Thompson Yates, will be opened next month by Lord Lister.

The composition fee for the college is £60, exclusive of certain special courses, and for the hospital practice £42.

### MANCHESTER.

Owens College: Medical Department.

The Royal Infirmary contains 300 beds, and has received 4,534 in-patients in the year. Associated with it are the convalescent home and the lunatic asylum.

The composition fee for the college is £70, and for the hospital practice £42.

### NEWCASTLE.

University of Durham: College of Medicine.

The Royal Infirmary, where the clinical instruction is given, contains 280 beds, about 4,043 in-patients being received during the year.

The composition fee for the college is £73 10s., and for the hospital about £30.

### OXFORD.

In connection with the University course, hospital practice may be seen at the Radcliffe Infirmary, which contains 120 beds. Clinical lectures in medicine and surgery, and demonstrations in pathology are given, as well as tutorial instruction.

### SHEFFIELD.

University College: Department of Medicine.

Clinical instruction is given in medicine and surgery at the Sheffield Royal Infirmary, which contains 240 beds, and the Royal Hospital, containing 125 beds, and students are also admitted to the Jessop Hospital for Diseases of Women, the City Fever Hospitals, and the South Yorkshire Asylum.

Composition fee for hospital practice £45, and for lectures and practical courses 60 guineas.

